# The gut microbiome as a plausible but unproven moderator of cinnamon trial outcomes in type 2 diabetes: toward phytochemical standardization and precision nutraceuticals

**DOI:** 10.3389/fnut.2026.1874182

**Published:** 2026-07-30

**Authors:** Eleje O. Okonta, Charles O. Nnadi, Ugwu Okechukwu Paul-Chima

**Affiliations:** 1 Department of Pharmacognosy and Environmental Medicine, Faculty of Pharmaceutical Sciences, University of Nigeria, Nsukka, Nigeria; 2 Department of Pharmaceutical and Medicinal Chemistry, Faculty of Pharmaceutical Sciences, University of Nigeria Nsukka, Nsukka, Nigeria; 3 Department of Research and Publication, Kampala International University, Kampala, Uganda

**Keywords:** *Cinnamomum*, glycemic control, gut microbiome, phytochemical standardization, polyphenols, precision nutraceuticals, type 2 diabetes mellitus

## Abstract

Cinnamon (*Cinnamomum* spp.) has been widely investigated as an adjunctive nutraceutical for glycemic management in type 2 diabetes mellitus, yet clinical findings remain inconsistent. This variability is commonly attributed to differences in cinnamon species, dosage, intervention duration, baseline glycemic status and phytochemical standardization, alongside methodological factors such as trial quality, dietary patterns, medication use, adherence and endpoint selection. One potential contributor that has received limited attention is the gut microbiome. We propose a testable hypothesis that a substantial proportion of the marked inter-trial heterogeneity observed in cinnamon meta-analyses (I^2^ > 75%) may reflect underlying gut-microbial metabotypes differing in their ability to convert cinnamon polyphenols and procyanidins into bioactive metabolites. Type 2 diabetes is associated with altered microbial composition, reduced butyrate-producing taxa and disrupted metabolic pathways. Cinnamon phytochemicals, including polyphenols, cinnamaldehyde, procyanidins and coumarin, undergo microbial biotransformation that may influence their bioavailability and metabolic effects. Because cinnamaldehyde is rapidly absorbed in the proximal gastrointestinal tract, colon-targeted delivery systems may be required to rigorously evaluate microbiome-mediated mechanisms. No randomized controlled trial has directly examined whether microbiome composition modifies cinnamon’s glycemic effects in type 2 diabetes. Future studies should therefore incorporate microbiome-informed designs, including phytochemical fingerprinting, safety monitoring and, where feasible, metagenomic and metabolomic profiling, to distinguish true biological non-response from intervention heterogeneity and advance precision nutraceutical approaches for diabetes management.

## 1 Introduction

Cinnamon (*Cinnamomum* spp.) has attracted sustained clinical interest as a low-cost adjunctive intervention for type 2 diabetes mellitus. Meta-analytic evidence suggests that cinnamon supplementation may improve fasting plasma glucose and selected lipid parameters, although effects on glycated hemoglobin remain inconsistent (1). A subsequent umbrella meta-analysis supports a possible glycemic benefit but also emphasizes substantial heterogeneity across interventional studies (2). Individual trials have reported neutral findings in some populations, including postmenopausal women with type 2 diabetes (3), whilst other syntheses indicate more consistent effects on fasting glucose than on glycated hemoglobin (4, 5).

This Perspective examines the gut microbiome as a plausible but empirically untested biological moderator of inconsistent cinnamon trial outcomes, with two specific aims. First, it synthesizes mechanistic and clinical evidence supporting the gut microbiome as a candidate determinant of individual variation in response to cinnamon-derived phytochemicals, while distinguishing human interventional evidence, microbiome association studies, *in vitro* and animal evidence, and hypothesis-driven reasoning wherever these differ. Second, it proposes a precision nutraceutical framework that integrates phytochemical standardization, microbiome profiling, paired metabolomics and structured glycemic phenotyping to address current gaps in trial design, while explicitly separating elements that are immediately implementable from those that remain aspirational. The intended contribution is a practical and scientifically grounded roadmap for advancing cinnamon research from population-level efficacy trials toward microbiome-stratified precision nutrition studies.

This Perspective is deliberately bounded in scope. It is not a systematic review of direct systemic cinnamon pharmacokinetics, and it is not a meta-analysis of cinnamon clinical efficacy; readers seeking exhaustive dose-response or trial-quality appraisal should consult the systematic reviews and meta-analyses cited above (1–5). The specific contribution offered here is a testable biological hypothesis, set out below, together with the trial-design architecture required to evaluate it.

The central, testable paradigm advanced in this Perspective is that individual responses to cinnamon supplementation can be provisionally stratified along a producer-to-non-producer gut-microbial metabotype gradient, defined by the capacity of an individual’s colonic microbiota to enzymatically convert cinnamon polyphenol and procyanidin matrices into bioactive small-molecule metabolites such as phenylpropionic acid derivatives. This framing is directly analogous to, although not yet empirically established for cinnamon, the producer and non-producer metabotypes described for other dietary polyphenols, most notably the equol-producer phenotype in isoflavone metabolism and the urolithin metabotypes described in ellagitannin metabolism (6). We propose this metabotype gradient, rather than a strict binary split, as the most parsimonious biological explanation compatible with the extreme heterogeneity (I^2^ > 75%) reported across cinnamon meta-analyses (1, 2), while explicitly acknowledging that this hypothesis has not been directly tested in any cinnamon trial and remains, at this stage, hypothesis-generating rather than established.

Explanations for mixed findings in cinnamon trials have most often focused on variation in cinnamon species, preparation type, dose, intervention duration and phytochemical standardization. These factors are important but incomplete. Substantial additional heterogeneity is likely to arise from trial quality (including randomization, blinding and risk of bias), baseline dietary pattern, concomitant medication use, particularly metformin, which independently alters gut microbial composition (7), adherence to the intervention, population and ethnic differences in baseline glycemic status, and inconsistent selection of glycemic endpoints across studies. These methodological and clinical factors should be regarded as primary, well-established candidate explanations for inconsistent findings. The gut microbiome represents a further, biologically plausible but as yet directly untested moderator that may act alongside, rather than instead of, these established sources of heterogeneity. Future studies should therefore move beyond the question of whether cinnamon lowers glucose on average and address which standardized preparation may benefit which microbiome-defined subgroup, through which metabolic pathway, at what safe dose, and only after known methodological sources of heterogeneity have been adequately controlled.

## 2 Gut microbial dysbiosis in type 2 diabetes and relevance to cinnamon metabolism

Type 2 diabetes is associated with reproducible alterations in gut microbial ecology, as shown in human metagenomic association studies. Qin et al. reported, in a metagenome-wide association study, reduced abundance of butyrate-producing bacteria and disrupted microbial functional pathways in individuals with type 2 diabetes (8). Karlsson et al. showed that gut metagenomic signatures differ across normal, impaired and diabetic glucose regulation (9), whilst Larsen et al. identified compositional differences between adults with and without diabetes (10). Subsequent work has confirmed reductions in genes involved in short-chain fatty acid biosynthesis and enrichment of pathways linked to branched-chain amino acid transport (7, 11). These are cross-sectional association studies and do not, by themselves, establish causality.

Mendelian randomization studies provide complementary, genetically-informed evidence and have identified candidate short-chain fatty acid-producing genera that may be causally linked to metabolic phenotypes (12). Host-microbiome interaction studies, largely derived from animal models and mechanistic human work, also indicate that microbial communities influence lipid and glucose metabolism through convergent metabolic and inflammatory pathways (13). Collectively, these observations provide a rationale, but not direct proof, for investigating the gut microbiome as a moderator of response to phytochemically complex botanical supplements such as cinnamon.

Cinnamon is not a single pharmacological entity but a complex botanical matrix. Its major bioactive constituents, including polyphenols, cinnamaldehyde, procyanidins and coumarin, may undergo variable pre-systemic microbial biotransformation in the colon, as demonstrated principally *in vitro* and *ex vivo* fermentation studies. Systemic exposure to biologically active metabolites therefore depends not only on preparation composition but also on the microbial enzymatic repertoire of the host. Participants receiving the same standardized cinnamon preparation may consequently experience different metabolic exposures according to microbial composition, enzymatic capacity, habitual diet, concomitant medication and intestinal barrier function; this remains a hypothesis-driven inference rather than a directly demonstrated clinical finding for cinnamon specifically. Integrative frameworks that combine phytochemical-based nutraceuticals with gut microbiota modulation have been proposed as a precision medicine approach for metabolic disease (14), yet cinnamon trials have not yet systematically adopted this paradigm, and no such trial has been conducted to date.

## 3 Microbial biotransformation of cinnamon phytochemicals

The relationship between dietary polyphenols and the gut microbiome is bidirectional, based largely on *in vitro* and mechanistic human evidence. Polyphenols may alter microbial community structure and function, whilst resident microorganisms transform polyphenols into metabolites with biological activities that differ from those of the parent compounds (15, 16). This biotransformation is mediated by enzymes such as deglycosylases, esterases, reductases and ring-fission oxygenases, the activity of which varies between individuals according to microbial community structure (17). Polyphenol bioavailability is also influenced by conjugation status, gastrointestinal matrix effects and microbial metabolism, yet cinnamon trials commonly report dose as total extract weight rather than as bioavailable exposure (18, 19). Habitual polyphenol consumption has been independently associated with distinct gut microbial profiles in observational cohort studies, further reinforcing the case for characterizing baseline dietary patterns and microbiome composition prior to intervention (20).

### 3.1 Upstream catabolism of cinnamaldehyde versus procyanidin matrices

Cinnamon phytochemicals do not share a common route of intestinal processing, and this distinction is central to interpreting inconsistent trial outcomes. Free monomeric trans-cinnamaldehyde is small, volatile and lipophilic; pharmacokinetic and toxicological evidence indicates that it is absorbed efficiently by passive diffusion across the gastric and proximal small intestinal mucosa, reaching peak plasma concentration within approximately 1–2 h of ingestion (21, 22). Under typical dosing, only a modest fraction of ingested cinnamaldehyde is therefore likely to reach the distal, bacterially dense colon intact, which constrains, rather than precludes, its direct engagement with colonic microbial biotransformation. This upstream absorption pattern implies that stimuli-responsive, colon-targeted encapsulation strategies, such as pH- or microbiota-triggered release matrices, would be required to test cinnamaldehyde-microbiome interactions directly, rather than assuming that orally administered cinnamaldehyde and colonic bacteria routinely interact *in vivo* at meaningful concentrations.

Cinnamon procyanidins and condensed tannins behave very differently from cinnamaldehyde: their higher molecular weight and polymeric structure limit upper gastrointestinal absorption, so that a substantial proportion reaches the colon largely intact, where microbial fermentation generates phenolic acids with potential insulin-sensitizing and anti-inflammatory properties (23). The cinnamaldehyde fraction that does reach the colon, together with any biliary-recirculated or partially metabolized material, may undergo microbial reduction and further catabolism to phenylpropionic acid derivatives, with systemic concentrations of these secondary metabolites partly determined by individual microbial metabolic capacity (16). These colonic metabolites may represent a substantial component of the host-relevant bioactive exposure, although the microbial communities responsible for their generation are rarely characterized in cinnamon supplementation trials.

**FIGURE 1:**
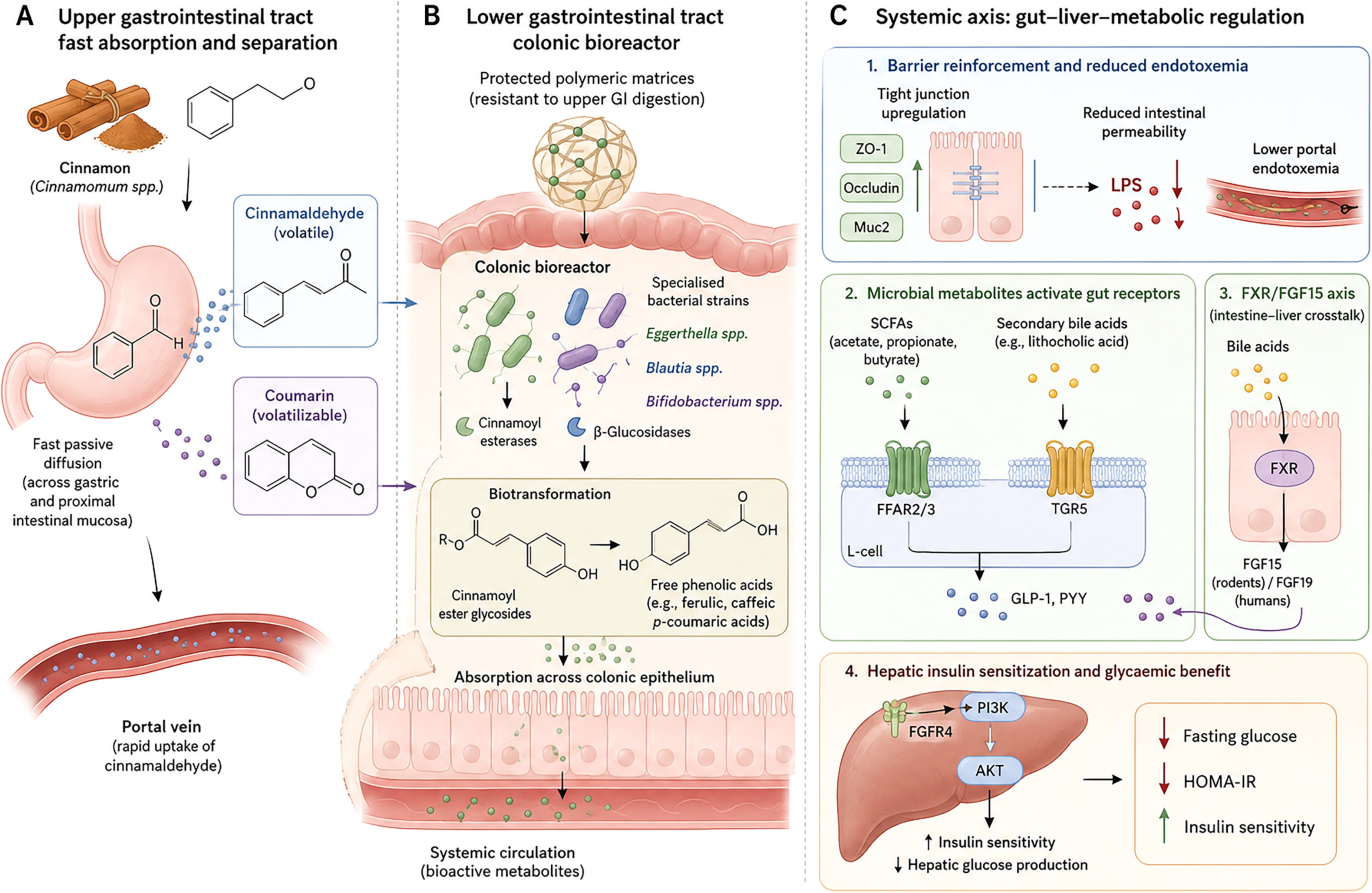
Cinnamaldehyde absorption kinetics and colonic bioreactor signaling to systemic glycemic pathways. **(A)** Upper gastrointestinal fast passive absorption and separation of volatile cinnamaldehyde and coumarin. **(B)** Lower gastrointestinal colonic bioreactor in which protected polymer matrices meet specialized bacterial strains (*Eggerthella, Blautia, Bifidobacterium*) expressing cinnamoyl esterases and β-glucosidases to yield free phenolic acids. **(C)** Systemic axis showing tight-junction upregulation (ZO-1, occludin, Muc2), reduced portal endotoxemia (LPS), SCFA/bile acid binding to FFAR2/3 and TGR5 receptors on L-cells, and the intestinal FXR/FGF15 axis triggering hepatic PI3K/AKT insulin sensitization.

A further consideration, established principally from *in vitro* antimicrobial screening, is that cinnamaldehyde possesses potent, broad-spectrum inter-kingdom antimicrobial activity. At the higher local concentrations achievable in the proximal gut after unformulated dosing, cinnamaldehyde can transiently suppress fiber-fermenting commensal taxa, with a plausible resulting reduction in colonic butyrate output. Cinnamaldehyde should therefore not be characterized as a straightforward, microbiome-nourishing prebiotic; its dual-edged kinetic profile, antimicrobial at the higher proximal concentrations reached after unformulated dosing, and potentially fermentable only when delivered in protected or attenuated colonic forms, should be made explicit in future trial rationale and formulation design (Figure 1).

### 3.2 Downstream receptor signaling via incretin modulation

Network pharmacology analyses, a computational and largely hypothesis-generating approach, have identified quercetin and related flavonoids as key cinnamon components with predicted affinity for antidiabetic targets including PPARG, INSR and AKT2 (24). These *in silico* predictions warrant cautious interpretation: such models typically evaluate binding potential without reference to achievable physiological concentrations, and parent cinnamon flavonoids are extensively modified by phase I/II hepatic and intestinal metabolism before reaching the systemic circulation, where they are present at low nanomolar to micromolar concentrations. Direct AKT2 engagement by intact parent compounds at these physiological concentrations remains unproven *in vivo*; any contribution of these predicted targets to cinnamon’s antidiabetic effects is therefore more plausibly mediated indirectly, through microbially generated metabolites and downstream receptor signaling, than through direct parent-compound binding (24).

Short-chain fatty acid production provides a mechanistic link, supported mainly by animal and mechanistic human physiology studies rather than cinnamon-specific trials, between cinnamon phytochemicals and host glucose metabolism. Fermentation of polyphenol-associated substrates and cinnamon-related oligomers may generate acetate, propionate and butyrate, which influence hepatic lipogenesis, intestinal gluconeogenesis, enteroendocrine signaling and insulin sensitivity (25, 26). SCFAs act through free fatty acid receptors (FFAR2 and FFAR3) on enteroendocrine L-cells to stimulate glucagon-like peptide-1 and peptide YY secretion, thereby modulating postprandial glucose excursions independently of pancreatic beta-cell function (27, 28). Diet-induced enrichment of SCFA-producing microbiota has been associated, in observational and dietary intervention studies unrelated to cinnamon, with improved glycemic control in type 2 diabetes (27, 29). Microbial bile acid metabolism provides a further pathway, as deconjugation and transformation of bile acids may activate farnesoid X receptor and Takeda G protein-coupled receptor 5 signaling, thereby modulating hepatic glucose output, incretin secretion and intestinal lipid handling (30, 31). Glucagon-like peptide-1 secretion is partly regulated by short-chain fatty acids and secondary bile acids (32, 33), providing an additional, mechanistically plausible route through which microbial composition may modify cinnamon-associated effects on postprandial glycaemia; direct evidence for this pathway specifically in cinnamon-treated humans is currently lacking.

## 4 Trial heterogeneity: Methodological noise or unmeasured responder Biology?

### 4.1 Established methodological sources of heterogeneity

Inconsistency in cinnamon trial outcomes has multiple plausible explanations, of which the gut microbiome is only one. Variation in trial quality, including randomization procedures, blinding and overall risk of bias, differences in baseline dietary pattern, concomitant medication use [particularly metformin, which independently alters gut microbial composition (7)], variable adherence to the intervention, population and ethnic differences in baseline glycemic control, and inconsistent selection of glycemic endpoints (fasting glucose versus glycated hemoglobin versus postprandial measures) collectively contribute substantially to observed heterogeneity. These well-established methodological and clinical factors are not superseded by the microbiome hypothesis advanced in this Perspective; rather, unmeasured responder biology, including microbiome-dependent phytochemical biotransformation, is proposed as an additional and complementary explanation that conventional trial designs have not been equipped to detect, and one that has not yet been directly tested in any cinnamon trial. Allen et al. reported favorable effects on fasting plasma glucose but not glycated hemoglobin (1), and Vanschoonbeek et al. found no significant glycemic improvement in postmenopausal women with type 2 diabetes (3).

### 4.2 Unmeasured responder biology and the producer/non-producer hypothesis

**FIGURE 2:**
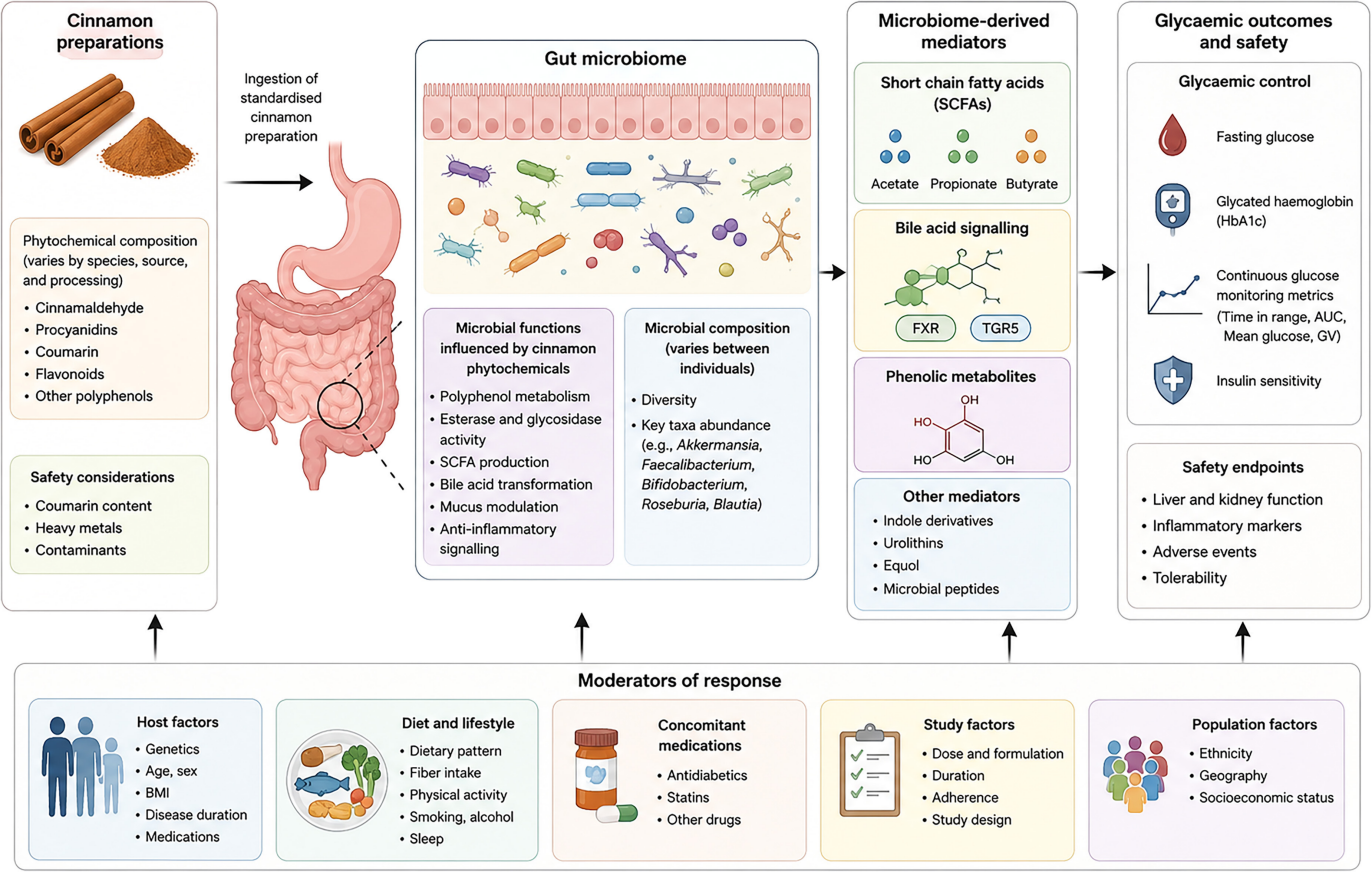
Gut microbiome mediated moderation of glycemic response to standardized cinnamon preparations in type 2 diabetes mellitus. Variability in cinnamon phytochemical composition and safety characteristics may influence gut microbial composition and functional activity, including polyphenol metabolism and short chain fatty acid production. Microbiome-derived metabolic mediators, including short chain fatty acids, bile acid signaling pathways and phenolic metabolites, may subsequently affect glycemic outcomes such as fasting glucose, glycated hemoglobin, continuous glucose monitoring metrics and safety endpoints.

In an observational precision-nutrition cohort study unrelated to cinnamon, Zeevi et al. showed that inter-individual variation in postprandial glycemic response is large, reproducible and partly predicted by gut microbiome composition even after adjustment for conventional dietary and clinical variables (34) (Figure 2). Diet-microbiota interactions are increasingly recognized, principally from observational and mechanistic studies, as important moderators of human metabolism more broadly (29, 35). It is therefore biologically plausible, although not yet empirically demonstrated for cinnamon specifically, that responses to phytochemically complex botanical interventions are similarly microbiome-dependent, and consistent with the producer/non-producer metabotype gradient proposed in Section “1 Introduction” (6). Polyphenol-microbiota cross-talk observed at a cardiometabolic level in other dietary contexts further supports this inference: individuals with distinct microbial profiles may generate different systemic exposures from identical polyphenol doses, which could plausibly contribute to heterogeneous metabolic outcomes across trials, although this specific mechanism has not been confirmed in cinnamon interventional studies (36).

## 5 From phytochemical standardization to precision nutraceuticals

Phytochemical standardization is necessary but insufficient for resolving cinnamon trial heterogeneity. Future studies should report species authentication, extract type, cinnamaldehyde content, polyphenol and procyanidin profiles, coumarin exposure and batch consistency; these are analytically well-established measurements that are readily achievable with existing chromatographic infrastructure and should be regarded as immediately implementable elements of trial design. Failure to distinguish *Cinnamomum cassia* from *Cinnamomum verum* introduces misclassification because these species differ substantially in coumarin concentration and relative phytochemical composition. Coumarin concentrations in commercial cinnamon products vary widely according to species and processing (21), and sustained intake of high-coumarin preparations may increase hepatotoxicity risk in susceptible individuals, particularly those with relevant CYP2A6-related variation (22).

A precision nutraceutical framework should integrate botanical, biological and clinical standardization. Botanical standardization ensures reproducible intervention composition and is immediately achievable. Biological stratification, which would identify participants whose microbiome, inflammatory state and metabolic phenotype may support efficient biotransformation of specific preparations, is conceptually attractive but currently more aspirational, since it depends on infrastructure and validated pipelines that are not yet standard in most trial settings. Clinical phenotyping, which confirms whether response is detectable across fasting, postprandial and longer-term glycemic endpoints, occupies an intermediate position and is largely feasible with existing clinical trial infrastructure. This tiered approach is consistent with precision nutrition research, which shows that dietary interventions exert context-dependent effects partly determined by individual microbiome composition (34, 35, 37), while acknowledging that translating this observation into cinnamon-specific trial designs remains to be demonstrated.

Paired stool and plasma metabolomics would provide a direct measure of cinnamon-derived metabolites reaching systemic circulation and would bridge the gap between administered dose and biologically active exposure; however, this remains an aspirational, resource-intensive addition rather than a near-term requirement for most trial settings.

Where paired metabolomics is undertaken, investigators should track a defined panel of target molecules in both plasma and stool rather than relying on non-specific volatile short-chain fatty acid measures alone: candidate targets include 3-(3-hydroxyphenyl)propionic acid, hippuric acid, ferulic acid sulfate and 4-hydroxybenzoic acid, alongside acetate, propionate and butyrate. Quantifying this panel provides a direct, molecule-level link between colonic biotransformation, described in Section “3 Microbial biotransformation of cinnamon phytochemicals,” and the systemic bioactive exposure that plausibly underlies cinnamon’s glycemic effects, rather than relying on microbial composition or SCFA concentration alone as indirect proxies.

Because polyphenol bioavailability is shaped by conjugation status, gastrointestinal transit and microbial enzymatic capacity (18, 19), these variables should ideally be characterized before standardized cinnamon preparations are advanced as precision nutraceuticals for metabolic disease, recognizing that this level of characterization may only be achievable in well-resourced research centers in the near term.

## 6 A microbiome-informed framework for future trials: feasible and aspirational elements

The individual elements of the framework proposed in this Perspective differ substantially in their current feasibility, and this distinction should guide how the framework is adopted in practice.

Immediately feasible elements. Species authentication using validated botanical and chemical methods, phytochemical profiling of cinnamaldehyde, polyphenols, procyanidins, coumarin and extract ratio by validated chromatographic methods, dose-response arms with objective adherence assessment, structured clinical phenotyping (insulin resistance indices, inflammatory biomarkers, hepatic function, dietary pattern and concomitant medication), and systematic safety monitoring of coumarin exposure, liver enzymes and renal function are all readily implementable using existing clinical trial infrastructure and should be prioritized as near-term additions to cinnamon trial design in most research settings.

Aspirational, omics-based elements. Baseline and longitudinal shotgun stool metagenomics, paired stool and plasma metabolomics (short-chain fatty acids, bile acids, phenolic acid metabolites and gut-derived inflammatory markers), and pre-specified microbiome-stratified subgroup analyses with causal mediation modeling require specialized sequencing, bioinformatic and statistical infrastructure that is not universally available, particularly in low- and middle-income country settings where the burden of type 2 diabetes is greatest. These components represent important longer-term goals rather than immediate requirements, and their implementation should be pursued through multicenter collaboration, shared infrastructure and international data-sharing initiatives rather than treated as a precondition for all future cinnamon research.

The confounding influence of metformin requires explicit operational handling rather than passive covariate listing. We recommend that future trial protocols adopt one of three approaches, selected according to feasibility and ethical constraints: (i) preferential recruitment of drug-naive cohorts where clinically appropriate; (ii) explicit stratification or matched randomization on stable metformin dose and duration where drug-naive recruitment is not feasible; or (iii) a medically supervised washout period prior to baseline microbiome sampling, undertaken only where withdrawal is ethically and clinically justified. Whichever approach is adopted, metformin exposure should be treated as a pre-specified stratification variable in the statistical analysis plan, rather than as a simple adjustment covariate, given its capacity to independently reshape both baseline microbiome composition and glycemic outcomes (7).

Statistical analysis plans for microbiome-stratified cinnamon trials must also account for the compositional nature of relative-abundance microbiome data, which does not conform to the assumptions of standard parametric tests. We recommend that future protocols pre-specify centered log-ratio (CLR) transformation of microbial abundance data prior to association testing, together with Benjamini-Hochberg false discovery rate correction for the resulting multiple comparisons (38). Building on this transformed feature set, we further propose an exploratory computational workflow in which baseline CLR-transformed microbiome vectors, functional KEGG Orthology pathway abundances, habitual dietary polyphenol intake scores and metformin dosing history are integrated within a supervised machine-learning architecture, such as a Random Forest or gradient-boosted (XGBoost) regressor, to generate a pre-intervention Cinnamon Responder Coefficient (CRc) (39). Analogous machine-learning frameworks applied to metagenomic feature sets have demonstrated reasonable discriminative performance for other disease phenotypes (39), but the CRc itself is a novel construct proposed here for future validation; it has not been tested in any cinnamon trial and should be treated as a hypothesis-generating stratification tool rather than a validated clinical instrument until prospectively evaluated.

Glycemic outcomes in future trials should include fasting glucose, glycated hemoglobin, postprandial excursions, standardized insulin sensitivity indices and, where feasible, continuous glucose monitoring-derived measures such as time in range and glycemic variability; these outcome measures are largely feasible with existing clinical infrastructure. Where the more aspirational omics components can be implemented, statistical analysis plans should pre-specify microbiome-stratified subgroup analyses and causal mediation models to help distinguish biological non-response from unmeasured heterogeneity, while trials lacking this infrastructure can still contribute meaningfully by rigorously implementing the immediately feasible elements described above. Table 1 summarizes the proposed design framework, and Table 2 provides corresponding multi-omic analytical specifications, together with an indicative feasibility tier for each domain.

**TABLE 1:** Proposed design framework for microbiome-informed cinnamon trials in type 2 diabetes mellitus.

Domain	Current limitation	Recommended approach	Scientific rationale	Feasibility tier
Botanical identity	Species reported generically or omitted	Authenticate *C. cassia*, *C. verum* or other *Cinnamomum* species by botanical and validated chemical methods	Reduces misclassification; species differ substantially in coumarin and cinnamaldehyde content (21, 22)	Immediately feasible
Phytochemical profile	Active compounds inconsistently reported across trials, without quantitative safety thresholds	Quantify cinnamaldehyde, polyphenols, procyanidins, coumarin and extract ratio by HPLC-MS/MS; standardize *C. verum* preparations to approximately 60%–75% trans-cinnamaldehyde and coumarin below 0.004% (≈40 ppm); explicitly contrast this with the substantially higher coumarin baseline (≈1.0%–1.5%) typical of *C. cassia*; track cumulative coumarin exposure against the EFSA tolerable daily intake of 0.1 mg/kg body weight	Improves reproducibility and safety; polyphenol bioavailability is matrix- and microbiome-dependent, and coumarin hepatotoxicity risk is species- and dose-dependent (18–22)	Immediately feasible
Dose and duration	Dosing broad and inconsistent; adequate duration rarely justified	Include dose-response arms with sufficient duration and objective adherence verification	Delineates therapeutic window; achievable systemic metabolite concentrations depend on microbial capacity (1, 2)	Immediately feasible
Host phenotype	Often limited to HbA1c and body mass index; metformin listed as covariate without operational handling	Characterize insulin resistance, inflammatory status, hepatic function, dietary pattern and concomitant medication; operationalize metformin exposure through drug-naive recruitment, stable-dose matching, or a medically supervised washout, and treat metformin status as a pre-specified stratification variable	Improves responder classification; metformin independently and substantially reshapes microbiome composition in type 2 diabetes (7)	Immediately feasible
Microbiome status	Gut microbiome not measured in the majority of trials; pipelines not standardized	Perform baseline and longitudinal shotgun stool metagenomics at a minimum depth of approximately 15–20 million paired-end reads per sample (40), using harmonized taxonomic (MetaPhlAn4) and functional (HUMAnN3) bioinformatic pipelines (41, 42) to ensure multi-center data compatibility	Identifies microbiome-defined responder phenotypes; microbial composition predicts glycemic response to dietary intervention (11, 34); adequate sequencing depth is required for stable taxonomic and functional resolution (40)	Aspirational (specialized infrastructure)
Microbial metabolites	Mechanistic metabolites rarely assessed	Measure paired stool and plasma SCFAs, bile acids, and targeted phenolic acid metabolites, including 3-(3-hydroxyphenyl)propionic acid, hippuric acid, ferulic acid sulfate and 4-hydroxybenzoic acid	Links cinnamon exposure to mechanism; SCFAs, bile acids and specific phenolic metabolites mediate glycaemic and incretin effects (25, 27, 28, 30, 32)	Aspirational (specialized infrastructure)
Glycemic outcomes	Reliance on fasting glucose and HbA1c alone	Include postprandial glucose, standardized insulin sensitivity indices and CGM-derived metrics (TIR, glycemic variability)	Captures dynamic glycemic response; CGM detects variability invisible to HbA1c (1, 34)	Largely feasible
Safety monitoring	Often underpowered and inconsistently reported	Monitor coumarin exposure, liver enzymes, renal function and adverse events systematically throughout the trial	CYP2A6-dependent coumarin hepatotoxicity risk varies by preparation type, dose and individual susceptibility (21, 22)	Immediately feasible
Statistical analysis	Conventional pooled-effect analyses only; subgroups not pre-specified; compositional structure of microbiome data not addressed	Apply centered log-ratio (CLR) transformation to compositional microbiome features, apply Benjamini-Hochberg FDR correction, and pre-specify microbiome-stratified subgroup analyses and causal mediation models in the statistical analysis plan	Distinguishes biological non-response from unmeasured heterogeneity; compositional data require CLR transformation for valid inference (38), and causal inference requires mediation modeling (12, 37)	Aspirational (requires microbiome data)
Predictive modeling	Responder heterogeneity is not predicted prior to enrolment; subgroups identified only *post hoc*	Integrate baseline CLR-transformed microbiome vectors, functional KEGG Orthology pathway counts, habitual dietary polyphenol scores and metformin dosing history into a supervised Random Forest or XGBoost regressor to derive an exploratory, pre-intervention Cinnamon Responder Coefficient (CRc)	Enables prospective stratification rather than *post hoc* subgroup discovery; comparable machine-learning architectures show reasonable discriminative performance on metagenomic feature sets (39), although the CRc itself requires prospective validation	Aspirational (requires prior multi-omic validation)

BMI, body mass index; CGM, continuous glucose monitoring; CRc, Cinnamon Responder Coefficient; FDR, false discovery rate; HbA1c, glycated hemoglobin; HPLC-MS/MS, high-performance liquid chromatography-tandem mass spectrometry; SCFA, short-chain fatty acid; TIR, time in range. Reference numbers correspond to entries in the numbered reference list. Domains are presented in the recommended sequence for protocol development. Feasibility tiers and proposed thresholds are indicative, intended to guide protocol development, and require prospective validation; they may vary by setting and available infrastructure.

**TABLE 2:** Multi-omic metric specifications for stratified clinical trials.

Analytical layer	Target metabolic parameter(s)	Minimum platform standard	Proposed stratification use
Metagenomic (taxonomic and functional)	Microbial community composition; KEGG Orthology functional pathway abundance	Shotgun sequencing at a minimum of 15–20 million paired-end reads per sample; taxonomic profiling with MetaPhlAn4 and functional profiling with HUMAnN3 (40–42)	Input feature vector for producer/non-producer metabotype classification and the Cinnamon Responder Coefficient (CRc)
Phytochemical fingerprinting	Trans-Cinnamaldehyde, coumarin, procyanidin and polyphenol content	HPLC-MS/MS; *C. verum* standardized to ≈60%–75% trans-cinnamaldehyde and coumarin < 0.004% (≈40 ppm); tracked against the EFSA tolerable daily intake of 0.1 mg/kg body weight (21, 22)	Confirms administered dose and exposure; gates trial safety monitoring
Fecal short-chain fatty acids	Acetate, propionate, butyrate	GC-MS as reference standard; targeted colorimetric or enzymatic proxy assay acceptable for resource-limited settings once validated against GC-MS	Mechanistic mediator measurement; candidate low-cost proxy marker for tiered translational pipeline
Paired plasma-stool targeted metabolomics	3-(3-Hydroxyphenyl)propionic acid, hippuric acid, ferulic acid sulfate, 4-hydroxybenzoic acid, secondary bile acids	Targeted LC-MS/MS panel with paired plasma and stool sampling	Confirms host-relevant bioactive exposure resulting from colonic biotransformation (Figure 1)
Statistical and computational layer	CLR-transformed microbial abundance features; BH-FDR corrected associations; exploratory CRc	Centered log-ratio transformation with Benjamini-Hochberg FDR correction (38); supervised Random Forest or XGBoost regressor (39)	Pre-intervention responder stratification; exploratory only, pending prospective validation

BH-FDR, Benjamini-Hochberg false discovery rate; CLR, centered log-ratio; CRc, Cinnamon Responder Coefficient; EFSA, European Food Safety Authority; GC-MS, gas chromatography-mass spectrometry; HPLC-MS/MS and LC-MS/MS, (high-performance) liquid chromatography-tandem mass spectrometry. All stratification cut-offs and the CRc construct are exploratory proposals intended to guide future protocol development and require prospective empirical validation; none has yet been tested in a cinnamon trial.

## 7 Discussion: limitations, future research priorities and translational considerations

It must be stated explicitly that no randomized controlled trial has yet tested microbiome-stratified cinnamon response in type 2 diabetes. The argument advanced in this Perspective is therefore hypothesis-generating and derived from indirect mechanistic, observational and association-based evidence rather than direct interventional data. This limitation is fundamental and should be borne in mind throughout: the gut microbiome is proposed here as a biologically plausible candidate moderator, not as an established determinant, of cinnamon trial outcomes.

Prospective randomized controlled trial evidence directly linking cinnamon-induced microbiome modulation to improved glycemic outcomes in type 2 diabetes remains entirely absent. The argument advanced in this Perspective is supported by human metagenomic association studies in type 2 diabetes (7–10), an observational precision nutrition study showing microbiome-associated glycemic variability in a non-cinnamon context (34), mechanistic evidence on polyphenol-microbiome interactions derived largely from *in vitro* and animal work (15–17), established principles of short-chain fatty acid and bile acid metabolism (25, 30, 31), and broader observational evidence that diet-microbiota interactions shape human metabolism (29, 35). None of this evidence directly demonstrates microbiome-dependent cinnamon efficacy in humans.

Several challenges must also be addressed before microbiome-stratified cinnamon trials can produce clinically actionable findings. Microbiome composition is sensitive to short-term dietary change, antibiotic exposure, medication use and technical variation in DNA extraction, sequencing and bioinformatic analysis (37). Background metformin therapy may independently alter gut microbial composition and could confound both baseline microbiome features and response to cinnamon supplementation (7). Inter-population microbial variation, shaped by long-term diet, geography, ancestry, early-life exposures and environmental factors, may also limit the generalizability of findings from single-center studies (35). As emphasized above, conventional methodological sources of heterogeneity, including trial quality, baseline diet, medication use, adherence, population differences and endpoint selection, must be adequately controlled in any future trial before microbiome-related variance can be meaningfully isolated.

Future research should prioritize adequately powered, multicenter randomized controlled trials that combine phytochemical standardization with rigorous dietary and medication covariate assessment as a first step, adding longitudinal metagenomics, paired metabolomics and pre-specified responder analyses where infrastructure permits. Harmonized analytical pipelines and open deposition of de-identified microbiome and metabolomic datasets would enable cross-study comparisons and future meta-analyses of microbiome-stratified subgroup effects. Such work would clarify whether cinnamon has clinically meaningful benefit in defined responder populations, a question that remains entirely open, rather than only modest or inconsistent effects in unstratified trial cohorts.

Translational implications and economic accessibility also deserve consideration. Cinnamon is cultivated predominantly in Asia-Pacific, South Asia and parts of Africa, making it broadly available and relatively affordable compared with pharmaceutical interventions for type 2 diabetes (43). This cost advantage is clinically significant given that the majority of individuals living with type 2 diabetes reside in low- and middle-income countries where access to specialist medicines and continuous glucose monitoring is constrained (44).

Reconciling cinnamon’s affordability with the cost of multi-omic characterization requires a deliberately tiered translational pipeline rather than uniform application of high-cost infrastructure across all settings. In this pipeline, shotgun metagenomics, untargeted LC-MS/MS metabolomics and continuous glucose monitoring would be deployed only within smaller, well-resourced discovery cohorts, with the explicit purpose of identifying low-cost proxy markers, such as targeted colorimetric or enzymatic SCFA assays, or quantitative PCR panels for a limited set of operational taxonomic units associated with producer status, that approximate the informative content of the full multi-omic signature. Once validated against the discovery-cohort multi-omic reference, these proxy markers, rather than the full sequencing and mass spectrometry infrastructure, could be deployed in subsequent pragmatic trials conducted in resource-limited public health settings, preserving cinnamon’s cost advantage for the populations that stand to benefit most while retaining biological grounding derived from the discovery-phase multi-omics.

However, the costs associated with metagenomics and metabolomics infrastructure required for the more aspirational elements of precision nutraceutical trials remain substantial, and these methodological demands are unlikely to be met within resource-limited health systems without international collaboration and open data-sharing. Standardized, low-cost phytochemical characterization protocols, which are immediately feasible, and the development of affordable SCFA biomarker panels, which remain aspirational, would together represent important intermediate steps in translating the precision nutraceutical model from high-income research settings to contexts where the type 2 diabetes burden is greatest. Ensuring that the clinical benefits of microbiome-stratified cinnamon research are accessible across income levels is therefore not only an ethical priority but also a scientific imperative if findings are to have global applicability.

## 8 Conclusion

Cinnamon research in type 2 diabetes should move beyond the binary question of average efficacy. A clinically relevant question for future research is whether specific phytochemically standardized cinnamon preparations produce measurable benefit in microbiome-defined responder populations, once established methodological sources of heterogeneity, including trial quality, baseline diet, medication use, adherence, population differences and endpoint selection, have been adequately addressed. The gut microbiome is a biologically plausible but currently empirically untested moderator of inconsistent clinical findings; no randomized trial has yet examined this question directly. Integrating microbiome science with botanical standardization, metabolomics, glycemic phenotyping and safety monitoring, including the producer/non-producer metabotype stratification and predictive responder modeling proposed above, could, if supported by future interventional evidence, help reposition cinnamon from a generic supplement toward a candidate precision nutraceutical for metabolic disease research. This framework, staged to distinguish immediately feasible from aspirational components, may also inform the evaluation of other complex botanical interventions whose clinical effects are hypothesized to depend on host microbial metabolism. Realizing the translational and public health potential of this framework will require sustained investment in low-cost genomic and metabolomic platforms, equitable access to precision nutrition infrastructure and multicenter collaborations that include populations from low- and middle-income countries where type 2 diabetes incidence continues to rise.

## Data Availability

The original contributions presented in this study are included in the article/supplementary material, further inquiries can be directed to the corresponding author.
